# Culturally Diverse Patient Experiences and Walking Interviews: A Co-Design Approach to Improving Organizational Health Literacy

**DOI:** 10.3928/24748307-20190828-01

**Published:** 2019-10-10

**Authors:** Jane Lloyd, Louise Dougherty, Sarah Dennis, Heather Attenbrow, Elizabeth Harris, Marilyn Wise, Mark Harris

## Abstract

People from diverse cultural and linguistic backgrounds are more likely to have low health literacy and less appropriate access to health services than other Australians. Interventions to improve health literacy have demonstrated moderate improvements in health service use. Most of these interventions focus on simplifying communication as opposed to navigation support. A comprehensive and multilevel response is required if the health care system and organizations are to be more responsive to different levels of health literacy. This includes obtaining feedback from patients on their experience of accessing health care. This study piloted the use of a co-design process to develop a culturally appropriate mechanism of elucidating the perspectives of community members of culturally diverse groups on their experiences of accessing a health service to identify the strengths and weaknesses of an organization's health literacy. This co-design process involved the adaptation of an existing “Walking Interview” tool to the location and language groups being targeted, as well as determining the process for recruiting participants and conducting the walking interviews. The interviews provided valuable insights into the experiences of culturally diverse groups in accessing Canterbury Hospital and identified areas for improvement, such as clearer signage and access to interpreter services. **[*HLRP: Health Literacy Research and Practice*. 2019;3(4):e238–e242.]**

The term health literacy can be broadly defined as the knowledge, skills, confidence, and networks that are necessary for staying healthy, accessing preventive screening, deciding on treatment options, self-management, and effective communication (Brega et al., 2015; Rudd, Comings, & Hyde, 2003). Health literacy is important to patients' health outcomes because it enables good patient-provider communication, self-management, and access to and use of health care (von Wagner, Steptoe, Wolf, & Wardle, 2009).

Low health literacy is unequally distributed among the population. People from diverse cultural and linguistic backgrounds and those who are experiencing socioeconomic disadvantage are more likely to have low health literacy (Taylor et al., 2017) and be less engaged with self-management (Ehrlich, Kendall, Parekh, & Walters, 2016). It is important not to conflate language barriers and health literacy. English-language barriers may be addressed through interpretation services. Health literacy barriers need to be addressed by simplifying communication, checking for understanding, navigation, and referral support (Institute of Medicine, 2012).

The attributes of the health care system and organizations play an important role in being responsive to people's health literacy and therefore improving access to care (Brach et al., 2012; Lloyd, Thomas, Powell-Davies, Osten, & Harris, 2018; Lloyd et al., 2018). The concept of a health literate organization was first proposed in a discussion article from the Institute of Medicine Roundtable on Health Literacy members in 2012 as a way of referring to an organization's efforts to support patient and community health literacy, as well as the organization's own cultural and community literacy (Brach et al., 2012).

Building organizational health literacy requires feedback from patients (Brach et al., 2012). To seek input from culturally and linguistically diverse groups, there is a need to think innovatively about how to obtain this feedback and involve patients in service redesign in a meaningful way. Bilingual Community Researchers (BCRs) are an emerging workforce that provides research support in languages other than English. This workforce can also act as cultural brokers, gathering feedback from people of culturally and linguistically diverse backgrounds on the health literacy of services and organizations.

The Canterbury area in New South Wales, located within Sydney Local Health District, has a high proportion of residents from diverse cultural and linguistic backgrounds who experience socioeconomic disadvantage (Dowsett & Broome, 2018). In Canterbury, 48% of residents were born overseas, compared to 33% in Greater Sydney as a whole (Van Buskirk & Broome, 2018). Every area within the Canterbury region has a higher than average proportion of low-income households compared to Greater Sydney (Van Buskirk & Broome, 2018).

This pilot study was conducted in Canterbury Hospital in 2017. The project was led by the Health Equity Research and Development Unit and was a collaboration between Canterbury Hospital, the University of New South Wales, the University of Sydney, and the Australian Commission of Safety and Quality in Health Care. The study aimed to explore the feasibility of using a co-design process with BCRs to identify the strengths and weaknesses of an organization's health literacy from the perspective of patients from culturally diverse backgrounds. This brief report will focus on how co-design was used in this study.

## Methods

A walking interview tool, adapted from the “Health Literacy Environment Packet: First Impressions and Walking Interview Tool” (Rudd, 2010) developed at Harvard University, was used in this study to guide BCRs in interviewing participants from culturally and linguistically diverse backgrounds. A co-design process was undertaken with the BCRs and the research team to tailor the walking interview tool for use in Canterbury Hospital and with the language groups being targeted (Arabic, Bengali, and Rohingyan). To adapt the tool, the wording was made specific to Canterbury Hospital (e.g., with references to specific locations within the hospital) and the questions were reworded into plain English. A consultation process with the BCRs was undertaken twice during the adaptation of the tool to ensure that it was culturally appropriate and that the language was clear. Emoji symbols and images were added to convey meaning more simply than through text. A brief health literacy screening question was added (“How confident are you filling in medical forms by yourself?”) (Chew et al., 2008) to establish the volunteer's approximate baseline health literacy.

The tool (Appendix 1) can be viewed at the following link: https://figshare.com/articles/Appendix_1-_The_Health_Literacy_Environment_Walking_interview_tool-_Canterbury_docx/9860678. The stages of the walking interview are outlined in **Table 1**.

### Staff and Training

Three BCRs were recruited to be involved in the design of the study and to conduct the walking interviews. The BCRs were funded by Sydney Local Health District. The languages spoken by the BCRs (in addition to English) were Bengali, Arabic, and Rohingyan. The Community Participation Coordinator based at Canterbury Hospital took on the role of study coordinator and gave dedicated time to the project to train and support the BCRs. This training involved the BCRs completing the walking interview in the role of the volunteer, with the Community Participation Coordinator in the role of the BCR.

### Recruitment and Walking Interview

The BCRs identified people within their community networks who might be interested in taking part in the study. They explained the study to them in the community member's native language and invited them to participate. After they had provided written consent, a time was made with each volunteer participant to go to Canterbury Hospital and meet the BCR to take part in the walking interview. BCRs conducted the walking interviews in their native language, with participants from local Arabic, Bengali, and Rohingyan-speaking communities.

When the participant arrived at the hospital, the BCR met them at the hospital café and purchased morning tea for them. This allowed the BCR and participant to develop some rapport and for the participant to feel more at ease in the environment. During morning tea, the BCR asked the participant the initial background questions from the Health Literacy Environment Walking Interview Tool and the Stage 1 questions about the participant's initial impressions of the hospital (Appendix 1). The interviews were audio-recorded by the BCRs and translated into written English with the help of the Community Participation Coordinator. BCRs also took notes on the form as the interview proceeded. After the background and Stage 1 questions were completed, the BCR then asked the participant to navigate his or her way to a particular service within the hospital (gestational diabetes unit or emergency department). Participants were not given a map or directions, as the purpose of the tool is not to test the participant's ability to navigate, but rather to find out how well the environment currently supports people to find their way around the hospital. As such, each participant was asked to find his or her way to the specified location using tools already present within the hospital (e.g., signage, asking staff for help, maps on the wall and so forth). The BCR accompanied the participant and asked questions along the way (see Stage 2 in Appendix 1). Once the participant reached the destination, the BCR then asked a series of questions about the participant's impression when they reached the destination as well as his or her impression when they entered the waiting room (see Stages 3 and 4 in Appendix 1). To conclude the interview, the BCR then asked the participant to reflect on the overall wayfinding exercise and to reflect on his or her previous experience communicating with health care providers (see Stage 5 in Appendix 1). Participants received a $50 shopping voucher at the conclusion of the interview as reimbursement for their time and travel expenses. The walking interviews were then translated, transcribed, and analyzed thematically.

### Ethics

Ethics approval to conduct the study was granted by the Sydney Local Health District-Royal Prince Alfred Hospital (RPAH) - Human Research Ethics Committee (HREC/16/RPAH/518). Participants gave their signed consent to participate in the walking interviews.

## Results

### Characteristics of Participants

Although the recruitment target was 12, only 9 participants of 21 completed walking interviews during the study period of April 2017 to July 2017. Five participants were women and four were men. Four spoke Arabic, three spoke Bengali, and two spoke Rohingyan. No pattern emerged by age or cultural background.

### The Walking Interview

The walking interviews lasted between 2 and 3 hours. The Community Participation Coordinator and the Lead Investigator read the transcripts and organized the feedback into the five walking interview categories (shown in **Table 1** in “Methods” section). The information was then synthesized into strengths and weaknesses of wayfinding at Canterbury. A meeting was held in September 2017 with the research team, the Community Participation Coordinator, and the BCRs to discuss the findings and to reflect upon what was learned about the feasibility of using the walking interview tool. Examples of major themes that emerged are presented in **Table 2**.

## Discussion

The role of health systems in meeting the needs of people from vulnerable groups, as well as reducing the health literacy demands on patients, is increasingly being recognized both within Australia and internationally (Brach et al., 2012; Institute of Medicine, 2012; Kindig, Panzer, & Nielsen-Bohlman, 2004; Nutbeam, 2008). This study piloted a method of using BCRs to co-design an intervention and to engage patients from culturally and linguistically diverse backgrounds to identify barriers to navigating health care services.

The walking interview provided valuable insights into accessibility and navigation issues at Canterbury Hospital. Although the interpretation of the findings of the study are limited by the small number of participants, this research demonstrates the feasibility of using a co-design process to adapt an intervention into different languages and to recruit and interview community members who may otherwise not be involved in strategic planning processes. Strengths (cultural responsiveness) and areas for improvement (such as clearer signage and access to interpreter services) were identified. Providing hospital tours in a variety of languages may be a beneficial way of increasing the confidence of community members in finding and using hospital services.

## Figures and Tables

**Table 1:** The Stages of the Walking Interview

1. First impressions when arriving at the hospital 2. Navigation and wayfinding 3. Impressions when destination reached 4. Observation of waiting room 5. Reflection on wayfinding exercise and previous experiences of communicating with health care providers

**Table 2:** Examples of Major Themes from the Walking Interviews

**Rating**	**Domain**	**Major Themes**
Areas of strength	First impressions	Positive atmosphere at entrance to hospital First impressions influenced (positively and negatively) by previous experiences at hospital
	Navigation and wayfinding	Participants preferred to ask staff rather than use a sign or a map Asking a staff member for help was a positive experience
	Arriving at a service	Either signage or staff members helped participants know they had reached their destination Many participants had a positive impression of the reception area
	Observation	Participants felt that there was sufficient space in the waiting room at the time they visited Easy to let staff know they had arrived
	Previous experience of communication between patients and health professionals (not specific to Canterbury Hospital)	Many participants felt positive about their previous interactions with health professionals and health services Participants felt that their questions were positively received and well-addressed by the health professionals Most participants reported that health professionals checked for understanding
Areas of further consideration	First impressions	Familiarity with the hospital brings confidence. One participant described feeling scared as this was the first time the person had been to the hospital, whereas participants who had been to the hospital many times described themselves as feeling confident Participants' previous experiences with hospitals form an unconscious bias that shape both positively and negatively the experience of accessing the hospital on future occasions
	Navigation and wayfinding	Participants noted there was no specific signage for the gestational diabetes mellitus (GDM) clinic. Participants were not always aware that the GDM clinic would be in the antenatal clinic
	Observation	Some participants commented that their English proficiency would impact on how easy or hard it was to let a staff member know they had arrived
Areas of attention and intervention	Navigation and wayfinding	Signs were in English only, which made it difficult for patients who did not read English and/or were unfamiliar with the Roman alphabetic script Maps were described as difficult to use/understand by many participants Participants (and staff) did not always differentiate between “diabetes” and “gestational diabetes,” which led to participants on occasion being directed to the wrong clinic (for example to the diabetes clinic rather than to the antenatal clinic)
	Arriving at a service	Although there was awareness by most participants of hospital interpreter services, the majority did not see any posters promoting the service
	Previous experience of communication between patients and health professionals (not specific to Canterbury Hospital)	More than one-half of respondents reported that waiting lists had caused difficulties in accessing care or services in the past. A small number of participants had difficulty accessing an interpreter service in their preferred language
